# Systematic review with meta-analysis of intraoperative neuromonitoring during thyroid reoperation

**DOI:** 10.12669/pjms.40.8.8241

**Published:** 2024-09

**Authors:** Shengwei Ji, Mingrong Hu, Chunjie Zhang, Maowei Pei

**Affiliations:** 1 Shengwei Ji Department of General Surgery, The People’s Hospital of Qingtian County, Zhejiang, 323900, China; 2 Mingrong Hu Department of Thyroid and Breast Surgery, The Affiliated Hospital of Hangzhou Normal University, Hangzhou, 310015, China; 3 Chunjie Zhang Department of General Surgery, The Zhejiang Hospital, Zhejiang, 310000, China; 4 Maowei Pei Department of General Surgery, The Zhejiang Hospital, Zhejiang, 310000, China

**Keywords:** Thyroidectomy, Thyroid reoperation, Thyroid Surgery, Recurrent laryngeal nerve, Intraoperative neuromonitoring

## Abstract

**Objective:**

Recurrent laryngeal nerve (RLN) injury is a serious complication during thyroid reoperation. Intraoperative neuromonitoring (IONM) is one of the means to reduce RLN paralysis. However, the role of IONM during thyroidectomy is still controversial. The aim of this study was to assess whether the IONM could reduce the incidence of RLN injury during thyroid reoperation.

**Methods:**

We performed a systematic review to identify studies in English language which were published between January 1, 2004, and March 25, 2023 from PubMed, EMBASE, and Cochrane Library, comparing the use of IONM and Visualization Alone (VA) during thyroid reoperation. The RLN injury rate was calculated in relation to the number of nerves at risk. All data were analyzed using Review Manger (version 5.3) software. The Cochran Q test (I^2^ test) was used to test for heterogeneity. Odds ratios were estimated by fixed effects model or random effects model, according to the heterogeneity level.

**Results:**

Eleven studies (3655 at-risk nerves) met criteria for inclusion. Data presented as odds ratio(OR) and their 95% confidence intervals(CI). Incidence of overall, temporary, and permanent RLN injury in IONM group were, respectively, 4.67%, 4.17%, and 2.39%, whereas for the VA group, they were 8.30%, 6.27%, and 2.88%. The summary OR of overall, temporary, and permanent RLN injury compared using IONM and VA were, respectively, 0.68 (95%CI 0.4-1.14, p=0.14), 0.82 (95%CI 0.39-1.72, p=0.60), and 0.62 (95%CI 0.4-0.96, p=0.03).

**Conclusions:**

The presented data showed benefits of reducing permanent RLN injury by using IONM, but without statistical significance for temporary RLN injury.

## INTRODUCTION

Thyroid reoperations can be challenging for surgeons because of the higher incidence of complications^1^ than in first performed thyroidectomy, particularly recurrent laryngeal nerve (RLN) injury.^2^ According to reported data, the incidence of RLN injury rate is higher in repeat than in first thyroid surgeries and it has been reported to approach 12.3 % for temporary injury and 3.8 % for permanent events.^3^,^4^ Unilateral RLN injury can lead to a variety of symptoms such as dysphonia, vocal fatigue, and dyspnoea, while bilateral RLN damage leads to airway obstruction.^5^ Thus, there is no doubt that RLN injury is a major cause of medical litigation and procedures to reduce the rate of RLN palsy have been the subject of investigation.

Routine exposure of RLN in thyroid resection has been considered the gold standard for preventing nerve injury for many years.^6^,^7^ However, during thyroid reoperation, it is sometimes difficult to visually to distinguish the nerve from the scar tissue and dissect the scar tissue or vessels surrounding the RLN.^8^ Recently, intraoperative neuromonitoring (IONM) has been introduced in order to identify or map the path of the nerves and to prevent their injury during surgery.^9^-^11^ However, the effect of IONM on RLN injury prevention in thyroid reoperation is still controversial. Some studies have shown that the use of IONM during thyroid reoperation decreased the rate of RLN injury,^12^,^13^ while others hold the opposite opinion.^14^-^18^ Due to the relatively small sample size, their conclusions have been questioned. In this study, we aimed to evaluate whether the IONM has an added advantages in decreasing RLN injury incidence during thyroid reoperation.

## METHODS

This systematic review was conducted by the Primary Reporting Items for Systematic Reviews and Meta-Analyses (PRISMA) Statement^19^ and the Cochrane Handbook for Systematic Reviews of Intervention. The study was approved by Hangzhou Normal University Affiliated Hospital Ethics Committee on December 11, 2019 (2019-HS-23). Studies comparing RLN injury rate incidence between IONM and VA during thyroid reoperation were retrieved from Pubmed, Medline, Embase, and Cochrane central register of clinical trials (CENTRAL). Publication data were selected from January 1, 2004, to March 25, 2023. We used the following free text search terms in “All fields”.

#1: “Thyroid Surgery” OR “Thyroidectomy” OR “Thyroid”
#2: “Intraoperative neuromonitoring” OR “Recurrent laryngeal nerve monitoring” OR “neuromonitoring”
#3: “RLN” OR “Recurrent laryngeal nerve” OR “nerve”

We used a combination of #1, #2 and #3 in literature search. There was no language restriction or methodological filters. Hand-searching of the reference list was performed in previous meta-analysis and relevant papers.

### Study selection

Two researchers independently read the title and abstract of the literature and screened the documents according to inclusion and exclusion criteria, then cross-checked. If there are differences between the two reviewers, the third evaluator to discuss and decide whether to include it or not. After obtaining the eligible studies, data were extracted into a predefined Excel table by one investigator and reviewed by another. Studies were included in the meta-analysis if they met the following criteria.

- Full English-language article on human patients.
- Any randomized control trial, prospective or retrospective comparative studies comparing the rate of RLN injury between thyroid reoperation with VA and IONM.
- Data on the number of RLN at risk and RLN injury that could be extracted from the published manuscript for calculation.
- Number of post-operative RLN injuries was determined by laryngoscopy.

Original articles that were pure descriptions of the methodology of IONM, animal studies, uncontrolled studies, case reports, expert opinions, and review articles without original data were excluded.

### Data collection

All data were extracted onto a standardized form. Primary data included type or design of the study, first authorship, country of study and dates, neuro-monitoring machine, electrode applied, stimulation current applied, site of nerve stimulation; and the data for calculating primary outcome separately by with and without IONM: number of patients, number of RLN at risk (NAR), definition and number of temporary and permanent RLN injury. The rate of RLN injury was calculated with the total number of NAR as the denominator. Temporary and permanent RLN injury was defined according to definition of the original article, and overall RLN injury was the sum of temporary and permanent injury.

### Statistical analysis

All individual outcomes were integrated with the meta-analysis software Review Manager Software 5.3. The odds ratios (OR) were used to reflect the association across studies. Statistical heterogeneity was calculated using Cochran Q. Heterogeneity was assessed with the I^2^ test by both fixed and random-effects model. Any I^2^ test > 50% was considered substantial heterogeneity. Fixed-effect models were used for analysis. If I^2^ test for fixed-effect models>50%, random-effects model was applied. Forest plots were generated to represent the OR with the corresponding 95% confidence intervals (CI) across the included studies. P-value < 0.05 was considered as statically significant.

## RESULTS

**Fig.1:**
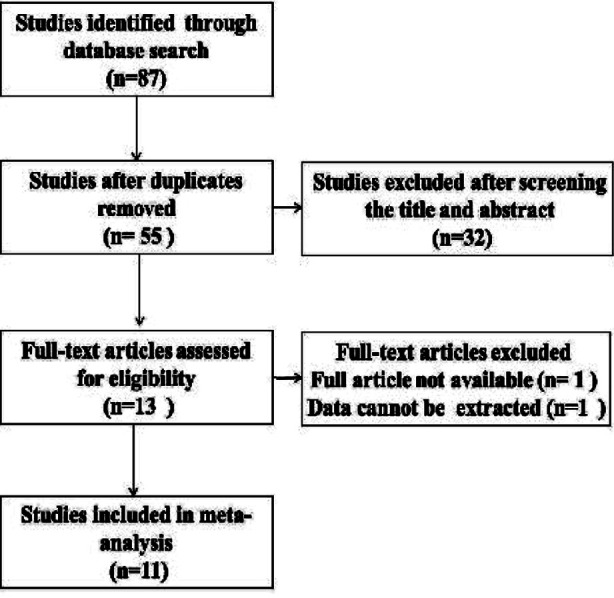
Flow diagram of article selection for inclusion in this study.

The electronic searches and review of the references yielded a total of 87 potentially relevant articles (Fig.1). All of them were published in English from 2004 to 2017 (Table-I). Table-I shows the key information such as definition of temporary and permanent RLN injury of the included study. Routine pre-operative and postoperative laryngoscopies were performed to evaluate vocal cord function in all studies. Permanent RLN injury was defined as RLN injury that did not recover within 12 months in four studies and six months in three studies. On the other hand, temporary RLN injury was defined as the recovery of RLN function within 12 months in four studies and six months in three studies. There were three studies which did not give any information about the definition of temporary or permanent RLN injury. In the study by Prokopakis et al, all patients with RLN injury recovered by four months.^17^ For IONM, insertion of needle electrodes into the vocal cord was reported in four studies, which all of them were at the early phase of studies. Surface electrode integrated into the endotracheal tube was used in five studies. Both needle electrodes and surface electrodes integrated into endotracheal tubes were used in two studies. Electrodes were connected to Neurosign 100 system (Magstim Clarify Company, Whitland, UK) in three studies, NIMS response 2.0 or 3.0 Nerve monitoring systems (Medtronic, Jacksonville, FL, US) in five studies, both Neurosign 100 system and NIMS response 2.0 or 3.0 Nerve monitoring systems were used in two studies, and NerveMonitor (Inomed, Germany) in one study. Stimulation current ranged from 0.05 to 5 mA. Pre-dissection vagal stimulations as suggested by a guideline from the International Neural Monitoring Study Group (INMSG) were performed in four studies. Both direct (RLN) and indirect (vagus) nerve stimulation were applied in seven studies.

**Table-I:** The characteristic in included studies.

Study (Year)	Country	Type of study	Sample size(total NAR)	Initial post-operative assessment		Definition of permanent RLN injury	IONM Set-up			Application of IONM			
	
				Time	Method		Electrode	Machine	Stimulation	Site of stimulation	Pre-dissection
Yarbrough(2004)^14^	USA	RCS	171	/	IL/FL	/	NE	/	/	RLN,Vagus	/
Chan(2006)^20^	Hong Kong	PCS	594	<2week	IL/FL	12 months	SE	Neurosign 100	0.5-1.5mA	RLN	/
Dralle(2008)^21^	Germany	PCS	4266	/	Laryngoscopy	6 months	NE	Neurosign 100	0.05-5mA	RLN	/
Alesina(2012)^18^	Germany	RCS	289	1 day	DL	6 months	before 2009,NE. After 2010,SE	Neurosign. NIMS 3.0	/	RLN,Vagus	/
Prokopakis(2013)^17^	Greece	RCS	121	/	FL		SE	NIMS	0.5mA	RLN	/
Barczynski(2014)^13^	Poland	RCS	1326	1 day	IL/DL	12 Months	2004-2007,NE. 2008-2012,SE	Neurosign 100. NIMS2.0/3.0	1mA,1mA	RLN,Vagus	YES
Calo(2014)^22^	Italy	RCS	2539	2 day	Laryngoscopy	12 months	SE	Nims2.0/3.0	/	RLN,Vagus	YES
Hei(2016) 23	China	RCT	84	1 day	Laryngoscopy	6 months	SE	NIM response2.0	/	RLN,Vagus	Yes
Chuang and huang(2013)^12^	Taiwan	RCS	85	/	Laryngoscopy	/	SE	NIM response system	1-2mA	RLN, Vagus	/
Wojtczak(2017)^24^	Poland	RCS	105	2 day	Laryngoscopy	12 months	NE	NIM3.0	1-2mA	RLN	
Jan Sopiński(2017)^25^	Poland	RCS	133	1 day	Laryngoscopy	/	NE	C2 NerveMonitor	1-2mA	RLN,Vagus	YES

***Abbreviation:*** NS: not stated; PCS: Prospective comparative study; RCS: Retrospective comparative study; RCT: randomized controlled trial; IONM: intra-operative neuro-monitoring. RL: rigid laryngoscopy; DL: direct laryngoscopy; IL: indirect laryngoscopy; FL: .exible laryngoscopy; SE: surface electrode at endotracheal tube; NE: needle electrode.

**Fig.2:**
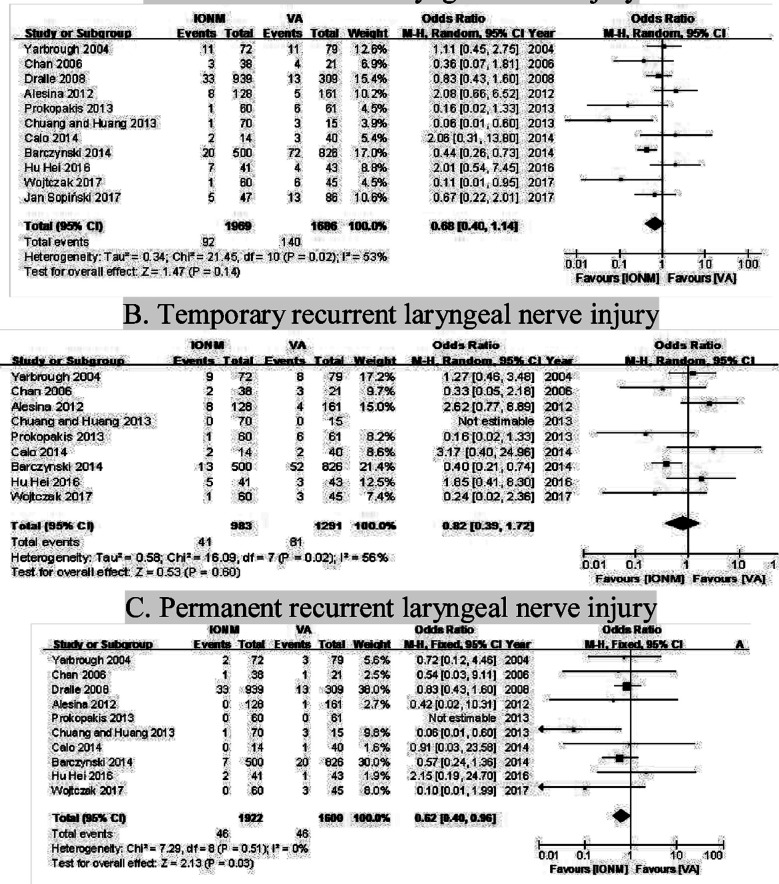
Forest plot showing the rate of (A) overall, (B) temporary and (C) permanent recurrent laryngeal nerve injury in intra-operative neuromonitoring (IONM) group and visualization alone (VA) group during re-operation thyroidectomy. ***Abbreviation:*** VA=visualization alone; IONM=intra-operative neuromonitoring.

According to data pooling from 11 studies, there were 3655 NARs in total, of which, 1969 NARs in the IONM group, and 1686 in the VA group. The overall, temporary, and permanent RLN injury rate, shown in Table-II, were 4.67% (n = 92), 4.17% (n=41) and 2.39% (n=46) respectively in IONM group compared with 8.30% (n =140), 6.27% (n = 81) and 2.88% (n =46) in VA group. The differences were statistically significant for permanent (p=0.03) but not for overall (p=0.14) and temporary RLN injury rate (p=0.60), which were shown in Fig.2.

**Table-II:** The main outcome in included studies.

Study(Year)	IONM	VA	IONM	VA	IONM	VA	IONM	VA

	Nerve at risk		Overall RLN injury		Temporary RLN injury		Permanent RLN injury	
Yarbrough(2004)	72	79	11	11	9	8	2	3
Chan(2006)	38	21	3	4	2	3	1	1
Dralle(2008)	939	309	33	13	/	/	33	13
Alesina(2012)	128	161	8	5	8	4	0	1
Prokopakis(2013)	60	61	1	6	1	6	0	0
Chuang and huang(2013)	70	15	1	3	0	0	1	3
Barczynski(2014)	500	826	20	72	13	52	7	20
Calo(2014)	14	40	2	3	2	2	0	1
Hei(2016)	41	43	7	4	5	3	2	1
Wojtczak(2017)	60	45	1	6	1	3	0	3
Jan Sopiński(2017)	47	86	5	13	/	/	/	/
Total	1969	1686	92	140	41	81	46	46
ncidence per NAR	/	/	4.67%	8.30%	4.17%	6.27%	2.39%	2.88%

The summary OR of overall RLN injury, temporary RLN injury, and permanent RLN injury for all included data for using IONM in comparison to visual identification alone on thyroidectomy in 11 studies, respectively, were 0.68 with 95% CI= 0.40 to 1.14 (p=0.02), 0.82 with 95% CI=0.39 to 1.72 (p=0.02), and 0.62 with 95% CI=0.40 to 0.96 (p=0.51). The presented data demonstrated a statistically significant difference between using IONM and visual identification alone for decreasing the permanent RLN injury rate. However, no significant differences were demonstrated for the overall and temporary RLN injury rate. The Cochrane Q test for heterogeneity indicated in temporary RLN injury (I^2^: 56.0%) and overall RLN injury (I^2^: 53.0%). Therefore, we adopted random-effect models in the analysis.

## DISCUSSION

Since its first introduction in 1966, IONM has been widely used in identify the RLNs during thyroidectomy.^26^ When performing potentially harmful procedures on the RLN, feedback from the neuromonitoring system guides the surgeon on whether to stop or change pathways, improving surgical technique and surgeons’ confidence.^27^ At the end of surgery, neurological tests performed by IONM can provide information about neurological function, which may influence surgical strategy as the procedure progresses.^28^ Once loss of signal on the dominant side of the surgical neck is confirmed, contralateral surgery should be stopped to avoid the risk of bilateral nerve injury.^29^-^32^ In addition, the IONM system can record the situation before and after thyroidectomy, which is very important for litigation. Despite the benefits of the technique, the role in revision thyroidectomy, particularly in preventing RLN injury or reducing the incidence of postoperative neurological injury, remain controversial. Numerous studies have shown that IONM is a promising neural identification tool to reduce the incidence of RLN injury during thyroid reoperation. Other studies have not shown a statistically significant advantage of IONM over VA in terms of overall, transient, or permanent RLN injury outcomes in thyroid reoperation. Therefore, our meta-analysis is appropriate to determine whether IONM plays a key role in thyroid reoperation.

Finally, our meta-analysis included 11 studies showing an overall rate of RLN injury for patients who underwent reoperations with IONM and VA were 4.67% and 8.30%. The OR was 0.68 with 95% CI= 0.40 to 1.14, suggesting that this monitoring approach prevents RLN injury during revision thyroidectomy. However, the difference is not statistical significance (p=0.14). To further investigate the role of IONM, we divided RLN damage into transient and permanent damage. Subgroup analysis did not reveal a statistically significant reduction in the frequency of temporary RLN changes (OR: 0.82, p=0.60). However, it has been observed that permanent damage to the RLN can be attenuated with IONM (OR: 0.62, p = 0.03). There are some reasons for this result. Due to the small sample size and possibly poor performance, we cannot deny or support the negative role of IONM in transient RLN disturbances. Due to the widespread use of total thyroidectomy, thyroid revision surgery was relatively infrequent, and the relatively small sample size was insufficient to demonstrate statistical significance.

Furthermore, while intermittent IONM is predictive of intraoperative and postoperative functional integrity of the RLN, it is recommended to avoid damage to the RLN during non-stimulation periods where damage has already occurred.^33^,^34^ In addition, IONM has enhanced the ability to see the anatomical details of scar tissue in the neck. The identification rate of RLNs by IONM was close to 100%. This allows IONM to localize RLNs prior to visual confirmation and helps distinguish RLNs from blood vessels and scar tissue.

Since IONM is used to identify RLNs, complete disconnection and permanent damage of RLNs has become less common. However, during thyroid revision surgery, clamping, stretching, electrothermal injury, and ischemia may cause temporary damage to the RLN. Therefore, IONM is more effective in preventing permanent RLN damage than temporary RLN damage. In addition, many studies did not assess whether revision surgery was performed on previously explored sites. These results may overlook the benefits of IONM. Future prospective controlled trials should focus on repeat surgery at previously operated sites to assess the utility of IONM.

### Limitations

We realize that there are some limitations in our review. First, only 11 studies met the inclusion criteria, some with small sample sizes. In addition, we cannot guarantee the accuracy of research methods, data, conclusions and other research results. Second, the heterogeneity was higher in the permanent RLN injury group than the transient RLN injury group. This may be related to differences in skill or experience levels of surgeons.

We know that the surgeon’s experience is the most important factor in the success of thyroid surgery. In addition, the risk factors such as type of disease, operation strategy, the number of previous operations, abnormal anatomy may also have different weights on the incidence of RLN injury. It should also be noted that different definitions of permanent RLN damage may affect our conclusions.

## CONCLUSIONS

This meta-analysis demonstrates no statistically significant difference in temporary and total RLN injury, but a statistically significant reduction in permanent RLN damage following the use of IONM in thyroid reoperation compared with VA group. We believe IONM is a promising tool that should be used routinely in thyroid reoperation.

### Authors’ contribution:

**MP:** Designed this meta-analysis and wrote the first draft of the manuscript; revising the paper; Submission the manuscript.

**SJ:** Extracted key data from the identified publications; revising the paper.

**MH**: Extracted key data from the identified publications; revising the paper.

**CZ:** Prepared Tables-I-II, Fig. 1 and 2, revising the paper.
